# Nanophthalmos

**Published:** 2011-04

**Authors:** Siamak Moradian, Azadeh Kanani, Hamed Esfandiari

**Affiliations:** Ophthalmic Research Center, Shahid Beheshti University of Medical Sciences, Tehran, Iran

A 45-year-old man with unremarkable past medical history presented with decreased vision following cataract surgery and piggy back intraocular lens (IOL) implantation in the right and left eyes, 5 and 3 weeks before respectively (Fig. 1). Calculated IOL power had been 50 D and 52 D in his right and left eyes respectively. Best corrected visual acuity was 20/200 in the right eye and 20/800 in the left one with 2+ relative afferent pupillary defect on the left side. Horizontal corneal diameter was 11.0 mm in both eyes. Keratometry was 50 D×15° and 45 D×105° in the right, and 51 D×135° and 50 D×45° in the left eye. Fundoscopic examination disclosed vascular tortuosity and choroidal effusion in both eyes together with exudative retinal detachment (ERD) in the left eye (Fig. 2). Echography revealed abnormal scleral thickening and choroidal effusion. Axial length was 14.2 mm and 14 mm in the right and left eyes respectively as determined by A-scan biometry. A diagnosis of nanophthalmos was entertained based on the above-mentioned findings. Choroidal effusion and ERD were assumed to be complications following cataract surgery.

## DISCUSSION

Nanophthalmos is a rare primary ocular disorder which usually occurs sporadically, however both autosomal dominant and recessive forms of inheritance have been reported.^1^ This condition is characterized by narrow palpebral fissures, a deep set globe in a small orbit, short axial length, a small hyperopic eye, and normal or reduced corneal diameter without gross structural defects.^2^ Histopathological studies have demonstrated scleral thickening and scleral collagen fiber abnormalities.^3^

The risk of complications during intraocular surgery in these patients is high and includes: shallow anterior chamber (AC), uveal effusion, cystoid macular edema, ERD, choroidal hemorrhage, malignant glaucoma, and vitreous hemorrhage.^4^,^5^ To prevent some of these complications, the surgeon should take special care to avoid hypotony during surgery and maintain AC volume throughout the procedure with abundant injection of viscoelastic materials.^6^ Small incision cataract surgery using phacoemulsification is therefore the method of choice; even without taking preventive measures such as iridotomy, iridectomy, laser iridoplasty or scleral incisions, the risk of complications is low. Careful preoperative assessment and appropriate precautions during surgery are crucial in these high risk patients.^5^

With current advances in the treatment of cataracts, glaucoma and uveal effusion, outcomes of surgery in nanophthalmic eyes is improving. However, complications may still arise, therefore unnecessary surgery should be avoided. Precise examination and timely intervention are necessary for proper management of adverse events.

## Figures and Tables

**Figure 1:**
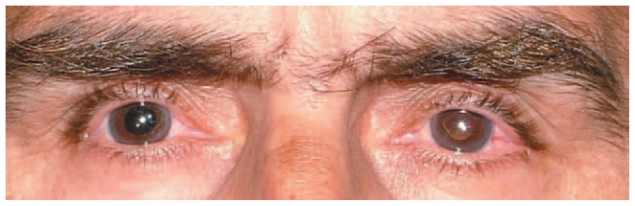
Gross appearance of the patient, note the narrow palpebral fissure and small deep set eyes.

**Figure 2:**
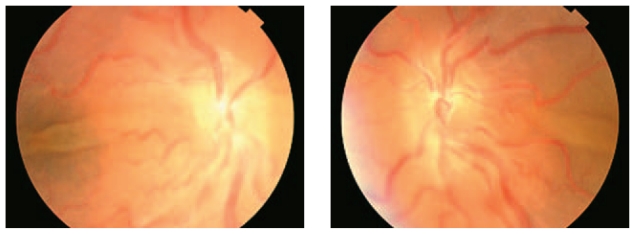
Exudative retinal detachment and vascular tortuosity in both eyes of the patient.
